# Bubbles in the barely born—contrast-enhanced ultrasound in neonates: a single-center experience

**DOI:** 10.1007/s00431-026-07166-0

**Published:** 2026-06-30

**Authors:** Alexandros Rahn, Thomas Müller, Diane Renz, Jens Drube, Doris Franke

**Affiliations:** 1 Department of Pediatric Pulmonology, Allergology and Neonatology, Hannover Medical School, Carl-Neuberg-Str. 1, 30625 Hannover, Germany; 2 Department of Pediatric Radiology, Institute of Diagnostic and Interventional Radiology, Hannover Medical School, Carl-Neuberg-Str. 1, 30625 Hannover, Germany; 3 Department of Pediatric Kidney, Liver and Metabolic Diseases, Hannover Medical School, Carl-Neuberg-Str. 1, 30625 Hannover, Germany

**Keywords:** Contrast-enhanced ultrasound, Neonates, Preterm infants, Point-of-care ultrasound, POCUS, Critical care, NICU

## Abstract

To assess clinical value, safety, and applicability of contrast-enhanced ultrasound (CEUS) in neonates, focusing on bedside use in intensive care. This retrospective single-center study included 23 neonates undergoing intravenous CEUS between 2010 and 2024, including seven preterm infants. The liver (*n* = 18) was the most frequently examined organ, followed by kidneys (*n* = 2), brain (*n* = 2), and adrenals (*n* = 1). Findings were compared with MRI (*n* = 10), CT (*n* = 3), or biopsy (*n* = 8), when available. In nine cases, CEUS findings were consistent with clinical course and treatment response only, without an independent reference standard. Among the 14 cases with available reference diagnostics, CEUS established the diagnosis first in ten (71.4%); histological confirmation was available in eight cases, with concordance in five (62.5%). 13 examinations (56.5%) were performed for bedside problem-solving in the neonatal intensive care unit, and no adverse events were observed.

*Conclusion*: CEUS provided clinically relevant diagnostic information in neonates, including preterm and critically ill infants and was well tolerated. CEUS was particularly valuable in hepatic vascular tumors, where it could provide a definite diagnosis, enabled hemodynamic assessment, supported treatment decisions, and aided identification of potentially life-threatening complications. Bedside use enabled timely diagnosis without sedation, intubation, or patient transport, thereby supporting minimal handling. In selected cases, CEUS may reduce the need for cross-sectional imaging. Despite off-label use, our findings suggest a beneficial role for CEUS in neonatal imaging, predominantly for hepatic indications.

**Table:** 

**What is Known:** • *Contrast-enhanced ultrasound (CEUS) is a radiation-free, bedside-compatible imaging technique with an established safety profile in pediatric patients.* • *Neonatal CEUS experience is limited to small case series, predominantly reporting cerebral applications.*
**What is New:** • *CEUS was feasible and well tolerated in neonates including preterm infants, with no adverse events in 23 examinations.* • *CEUS yielded clinically relevant diagnostic information across a range of indications, primarily hepatic, encompassing both focal lesions and perfusion assessment, supporting clinical decision-making in neonatal care.*

**Supplementary Information:**

The online version contains supplementary material available at 10.1007/s00431-026-07166-0.

## Introduction

Ultrasound (US) is the first-line imaging modality in neonatal and pediatric patients because it is widely available, allows bedside imaging, and does not involve ionizing radiation [1]. In neonates, especially preterm infants, these characteristics are crucial, as diagnostic procedures should minimize handling, transport, sedation, and stress [2]. When conventional US findings remain inconclusive, additional imaging such as MRI or CT is often needed [3]. MRI may necessitate sedation or general anesthesia, and CT involves ionizing radiation. Moreover, both modalities require transport of the neonate out of the neonatal intensive care unit (NICU) or the general ward and are therefore risky for the sick infant and time-consuming for staff [4, 5].

Contrast-enhanced ultrasound (CEUS) enables real-time assessment of tissue perfusion without radiation, sedation, or nephrotoxic contrast agents using intravascular microbubble ultrasound contrast agents (UCA). The UCA remains strictly intravascular, depicts the microcirculation due to its small microbubble size (< 6 μm), and is rapidly eliminated, with the gas core exhaled via the lungs and the shell metabolized by the liver [6]. The microbubbles oscillate and lead to resonant and enhanced backscattering of US signals, allowing dynamic visualization of even tiny vessels [7]. Clinical practice guidelines exist for CEUS use in adults [8, 9], and experience with CEUS in pediatric imaging has increased recently [10–15].

In Europe, the second-generation UCA SonoVue® (Bracco) is widely used, although intravenous CEUS remains off-label for pediatric indications [16]. In the United States, the identical UCA is marketed as Lumason® and is approved for intravenous liver imaging in pediatric patients [17]. Previous studies report a low incidence of adverse reactions following CEUS in children, with mild reactions occurring in ∼1% and serious adverse events in about 0.2% [18, 19], with rates slightly higher than in adults [20].

Clinical experience with intravenous CEUS in neonates remains limited, and studies are still lacking. Most reports focus on cerebral perfusion imaging [21–24], while data on other organ systems are scarce. We therefore report our single-center experience in neonates, assessing feasibility, diagnostic value, and safety, with focus on extracerebral applications and bedside use in intensive care settings.

## Methods

This retrospective single-center study was approved by the local ethics committee (No. 12340-BO-K-2026), and written informed consent for the off-label use of the UCA was obtained from all legal guardians. The principles of the Declaration of Helsinki were taken into account.

The study included all neonates who underwent intravenous CEUS at our tertiary pediatric center between 2010 and 2024 within the first 28 days of life. Each patient had a single examination; one intracavitary CEUS examination was excluded. Examinations were categorized according to clinical setting (NICU vs general ward) and urgency. Examinations were classified as clinically urgent when CEUS was requested due to acute clinical deterioration or a time-sensitive diagnostic question.

### Ultrasound equipment and contrast agent administration

All US examinations were performed by a single examiner with longstanding experience in pediatric US and CEUS (DEGUM Level III). High-resolution US systems included Toshiba Aplio XG (Toshiba Corporation) and GE Logiq S8, E9, and E10 (GE Healthcare Technologies Inc.). Examinations were conducted using linear and convex transducers (PLT-850AT, PVT-375BT, 9L, C2-9, and exceptionally C1-6). Each examination started with conventional B-mode and Doppler sonography, followed by CEUS using manufacturer-specific contrast presets with a low mechanical index. Imaging depth and sector width were restricted to the target region, and gain settings were adjusted close to the noise threshold to suppress background signal and optimize microbubble visualization [7]. SonoVue® (sulfur hexafluoride, SF₆) was used and administered intravenously via a ≤ 24G peripheral cannula (*n* = 17) or central venous catheter (*n* = 6). 28G peripherally inserted central catheters (PICCs) were avoided due to microbubble destruction. After injection, up to 5 ml of saline was used for flushing. All CEUS cine loops were stored in the institutional picture archiving and communication system (PACS). Infants were continuously monitored for heart rate and oxygen saturation as part of routine neonatal care. Following the examination, blood pressure was measured at regular intervals for at least 60 min.

### Reference diagnostics and comparative procedures

When available, CEUS findings were compared with MRI, CT, or biopsy. All imaging data, including CEUS findings and reference diagnostics, were independently and blindly reviewed by two experienced clinicians (DF, AR); AR had no prior knowledge of CEUS findings or clinical outcome, and discrepancies were resolved by consensus discussion. Temporal order of diagnoses was established based on documented examination dates from the institutional PACS and medical records. These findings were then categorized according to predefined diagnostic certainty criteria: a “diagnosis” denoted a definitive, clinically valid interpretation. “Suspected (correct)” referred to a presumptive diagnosis subsequently confirmed by another imaging modality, histopathology, or clinical course. “Suspected (incorrect)” referred to a presumptive diagnosis that was later refused. “Descriptive” findings were visualized but did not allow a definitive conclusion. To assess the diagnostic contribution of each modality, the concept of “diagnosis first by” was applied, defined as the earliest correct interpretation that remained unchanged. If a modality raised a correct suspicion later confirmed by clinical course or treatment response, it was still counted as first, unless another modality had already provided a definitive diagnosis.

### Statistical analysis

Data were analyzed descriptively using GraphPad Prism 10.4.1 (GraphPad Software). Normality was assessed using the Shapiro–Wilk test. Normally distributed data were presented as mean ± standard deviation (SD), while non-normally distributed data were given as median and interquartile range (IQR).

## Results

### Study population and clinical characteristics

23 neonates (13 male, 10 female) were included in the final analysis (Supplemental Fig. 1). Seven were born preterm with a median gestational age of 33 ^6^/_7_ weeks (range 31 ^2^/_7_–35 ^5^/_7_), while the overall median gestational age was 38 ^3^/_7_ weeks (range 31 ^2^/_7_–41 ^3^/_7_). CEUS was performed at a median postnatal age of 12 days (range 1–28). 13 examinations (56.5%) were performed in intensive care units, and seven (30.4%) were conducted under urgent conditions. The most frequent indications were evaluation of unclear hepatic lesions, acute liver failure, suspected congenital tumors, perfusion assessment, and screening for hepatic hemangiomatosis in infants with multiple cutaneous hemangiomas. The liver was the most commonly examined organ (18/23), followed by kidneys (2/23), brain (2/23), and adrenal region (1/23). The median initial weight-adjusted UCA dose was 0.44 ml/kg. In 14 cases (60.9%), a second bolus was administered (median 0.36 ml/kg). Demographic, procedural, and diagnostic data are summarized in Table 1, Supplemental Table 1, and Supplemental Fig. 2.

**Table 1:** Demographic and clinical characteristics of the study population

Characteristics	Value*
Number of patients/examinations	23/23
Sex	Female: 10 (43.5%) Male: 13 (56.5%)
Gestational age (p.m., weeks)	38 ^3^/_7_ (35 ^2^/_7_–39 ^6^/_7_) Range: 31 ^2^/_7_–41 ^3^/_7_
Birth weight (kg)	3.04 ± 0.76 Range: 1.21–4.32
Birth length (cm)	48.85 ± 4.41 Range: 40–55
Age at examination (days)	12 (2–23) Range: 1–28
Weight at examination (kg)	3.15 ± 0.80 Range: 1.4–4.6
Length at examination (cm)	49.26 ± 4.47 Range: 40–56
First intravenous UCA dose (ml), *n* = 23	1 (0.9–1.9) Range: 0.5–2
First intravenous UCA dose (ml/kg), *n* = 23	0.44 (0.23–0.60) Range: 0.17–1.07
Second intravenous UCA dose (ml), *n* = 14	1 (0.85–2) Range: 0.6–3
Second intravenous UCA dose (ml/kg), *n* = 14	0.36 (0.28–0.56) Range: 0.17–1.07
Lesion size (cm), *n* = 18	5.58 ± 3.52 Range: 0.9–13.2

*Non-normally distributed data are shown as median (interquartile range), whereas normally distributed data are presented as mean ± SD. p.m.: post menstruationem, UCA: ultrasound contrast agent

### CEUS findings

CEUS enabled real-time assessment of lesion vascularity and organ perfusion, and revealed a broad spectrum of vascular, neoplastic, and perfusion-related abnormalities.

**Fig. 1:**
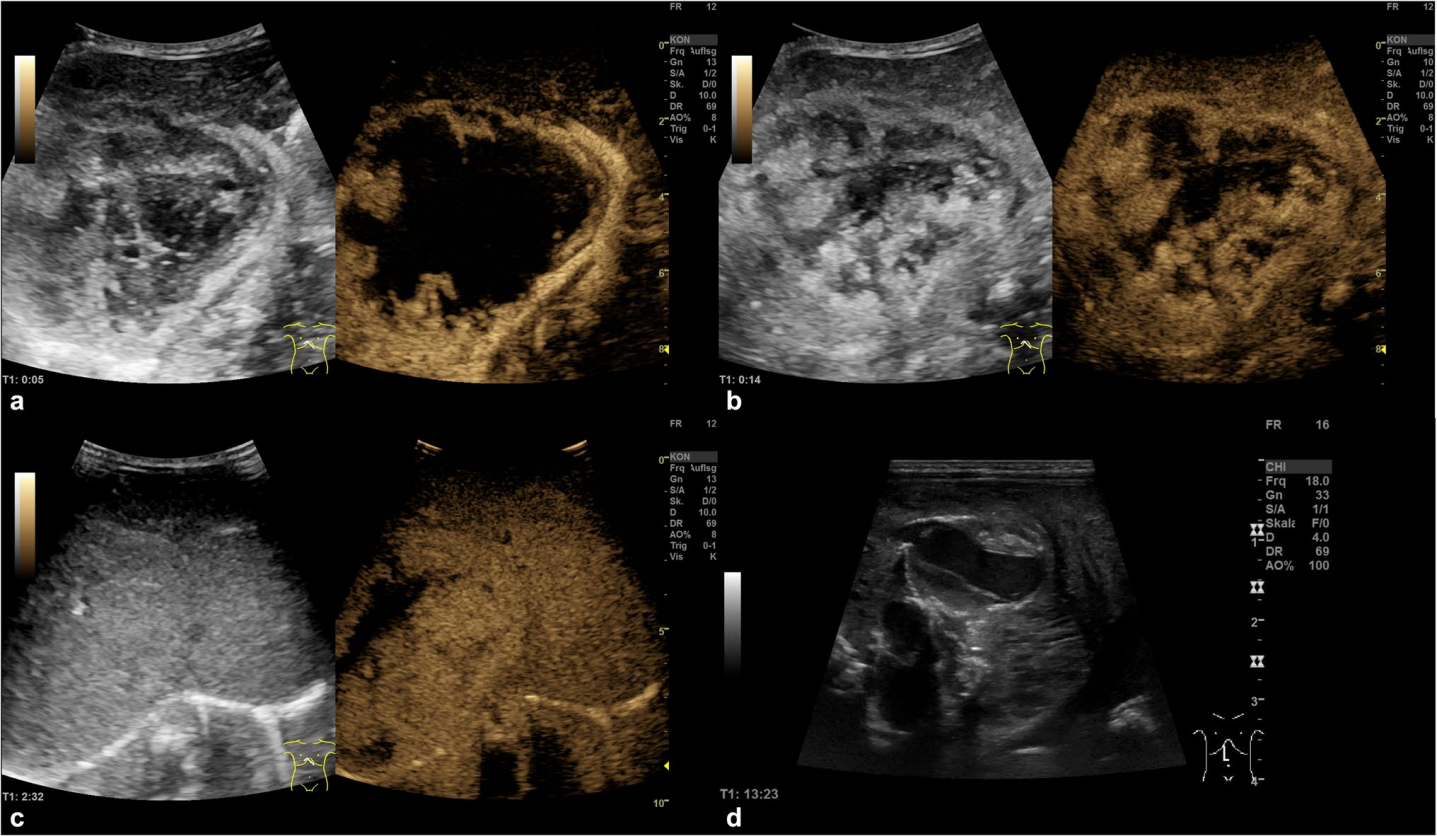
Giant congenital hepatic hemangioma in a neonate born at 39 ^3^/_7_ weeks’ gestation, investigated on the second day of life under high-flow respiratory support (weight 4.32 kg, length 53 cm). Heterogeneous lesion in segments V–VIII, measuring 10 × 9 × 5 cm (∼220 mL). Highly elevated hepatic artery flow velocity 232/109/0.53 (systolic/diastolic/RI). Coiling of two of three arterial feeder vessels was performed on the seventh day of life, followed by off-label sirolimus therapy due to respiratory deterioration. **a** Subcostal view. CEUS in the early arterial phase (5 s after ultrasound contrast agent injection; UCA). Centripetal contrast enhancement in the peripheral parts of the vascular tumor which affects nearly the whole right liver lobe. Early non-enhancement in the center. Nodular rim enhancement similar to infantile low-flow hemangiomas, compare Fig. 2. **b** Only a few seconds later increased centripetal enhancement in the inner layers. Yet non-enhancement in the more central parts. **c** Portal venous phase 2 min 32 s after UCA injection. Further slow centripetal fill-in, still with non-enhancement in the central core (“iris diaphragm phenomenon”). **d **High-frequency B-Mode image of a partly thrombosed giant vessel within the giant congenital hepatic hemangioma, illustrating the high risk of potentially lethal Kasabach-Merritt phenomenon in these patients

**Fig. 2:**
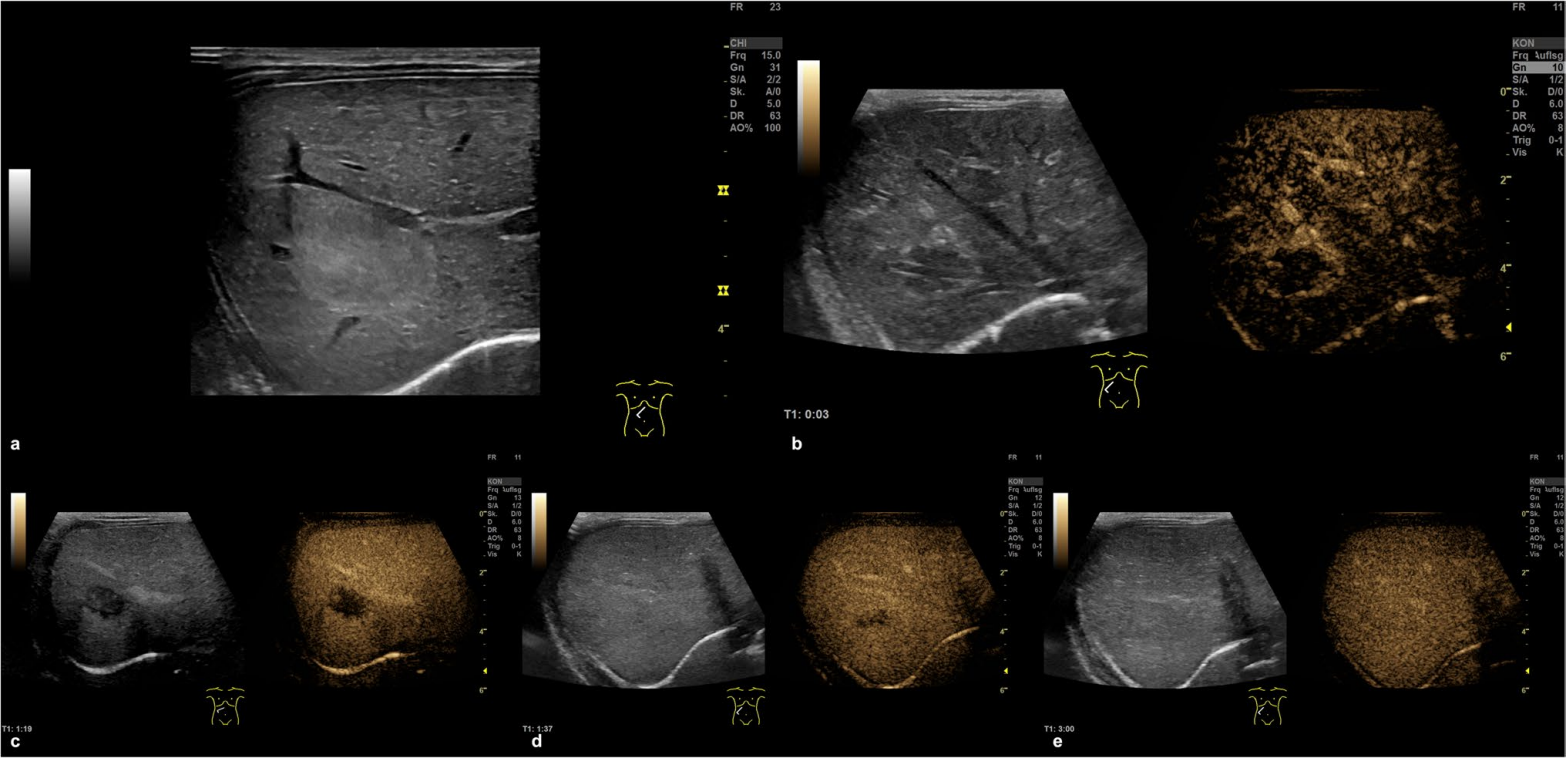
Infantile single low-flow hepatic hemangioma in a neonate born at 40 ^1^/_7_ weeks’ gestation, identified incidentally on the 11th day of life (weight 2.7 kg, length 50 cm), who was transferred to our clinic. **a** Subcostal view.B-Mode image of an echogenic, roundish, and sharply delineated lesion in the right liver lobe of 2.4 × 1.7 × 1.9 cm. Even with high sensitivity Doppler or microvascular imaging no vessels could be detected in the lesion (not shown). **b **Early arterial phase, 3 s post-injection: nodular rim enhancement in the periphery of the lesion, early non-enhancement of the center. **c **Portal venous phase, 1 min and 19 s post-injection: nodular rim enhancement in the lesion, slow centripetal fill-in. **d **Portal venous phase, 1 min and 37 s post-injection: slow further centripetal fill-in within the lesion. **e **Late phase, 3 min after post-injection. Complete fill-in in the whole lesion, isoenhancement of the lesion compared with the liver parenchyma, no wash-out. Typical finding for an infantile low-flow hemangioma, which is far less common in infants than infantile high-flow hemangiomas (“iris diaphragm phenomenon”). Because of the classical finding no further diagnostic imaging or biopsy was needed. Spontaneous regression in follow-up scans

**Fig. 3:**
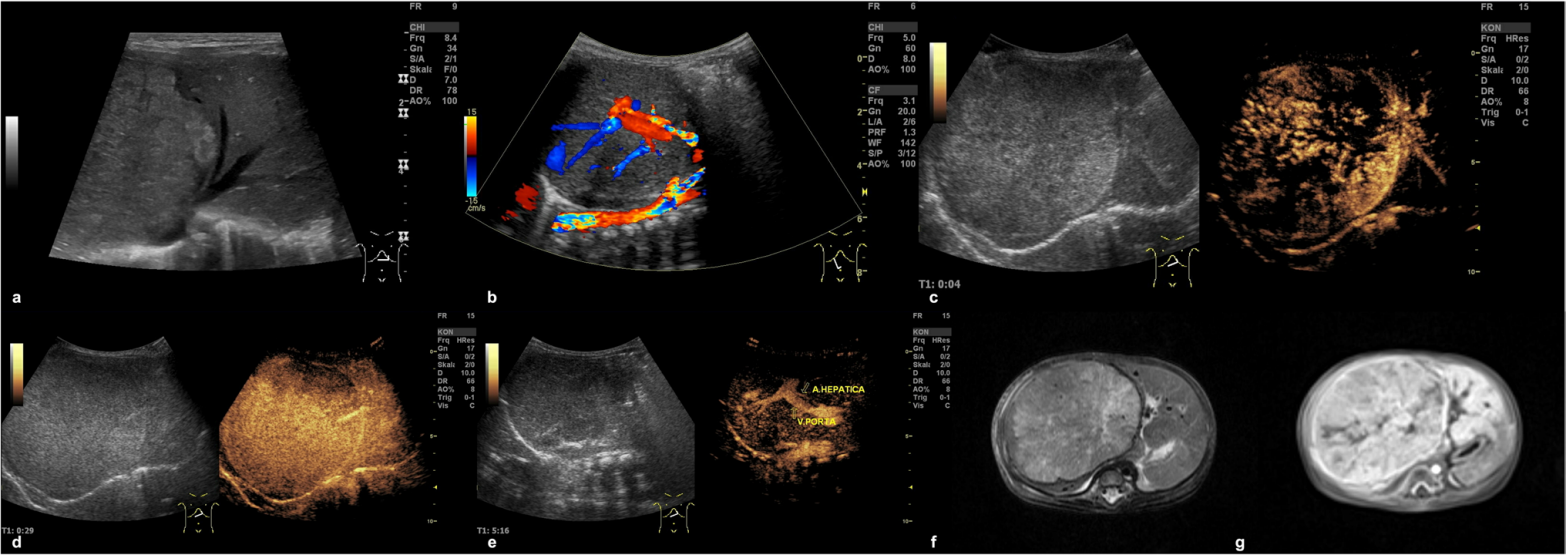
Hepatoblastoma in a neonate born at 41 ^1^/_7_ weeks’ gestation, presented on the ninth day of life (weight 3.46 kg, length 52 cm). Tumor in the whole right liver lobe (segments V–VIII), measuring 9.2 × 8 × 9 cm (~ 330 mL). AFP markedly elevated at 1.370574 µg/L (normal < 41.687 µg/L). The patient received three cycles of cisplatin-based chemotherapy followed by right hemihepatectomy, resulting in sustained remission. **a** Subcostal view. B-Mode image through the right and left liver lobe showing an echorich, well-delineated tumor within the whole right liver lobe obstructing the right liver vein. No portal venous branches can be seen within the tumor. The parenchyma of the left liver lobe is normal. **b** Doppler scan in an oblique-longitudinal section through the liver vessels and the aorta. Marked increase of the diameter and hepatic artery flow due to tumoral needs. **c** In the early arterial phase (4 s post-injection), CEUS shows multiple tortuous tumor vessels appearing at the same time in the periphery and the center of the tumor. This underlines again the high diagnostic value of the early arterial phase. **d** In the beginning of the portal venous phase of CEUS the hepatoblastoma is now completely enhanced. **e** CEUS 5 min and 16 s post-injection, subcostal oblique view. Marked washout within the hepatoblastoma in the right liver lobe, but still good enhancement in the portal vein and the hepatic artery. No signs of vessel infiltration or tumor thrombosis. **f** Axial T2-weighted MRI with fat suppression showing a liver tumor with heterogeneous structure and minimal central necrosis. **g** Axial post-contrast T1-weighted MRI with fat suppression demonstrating marked contrast enhancement of the liver tumor

**Fig. 4:**
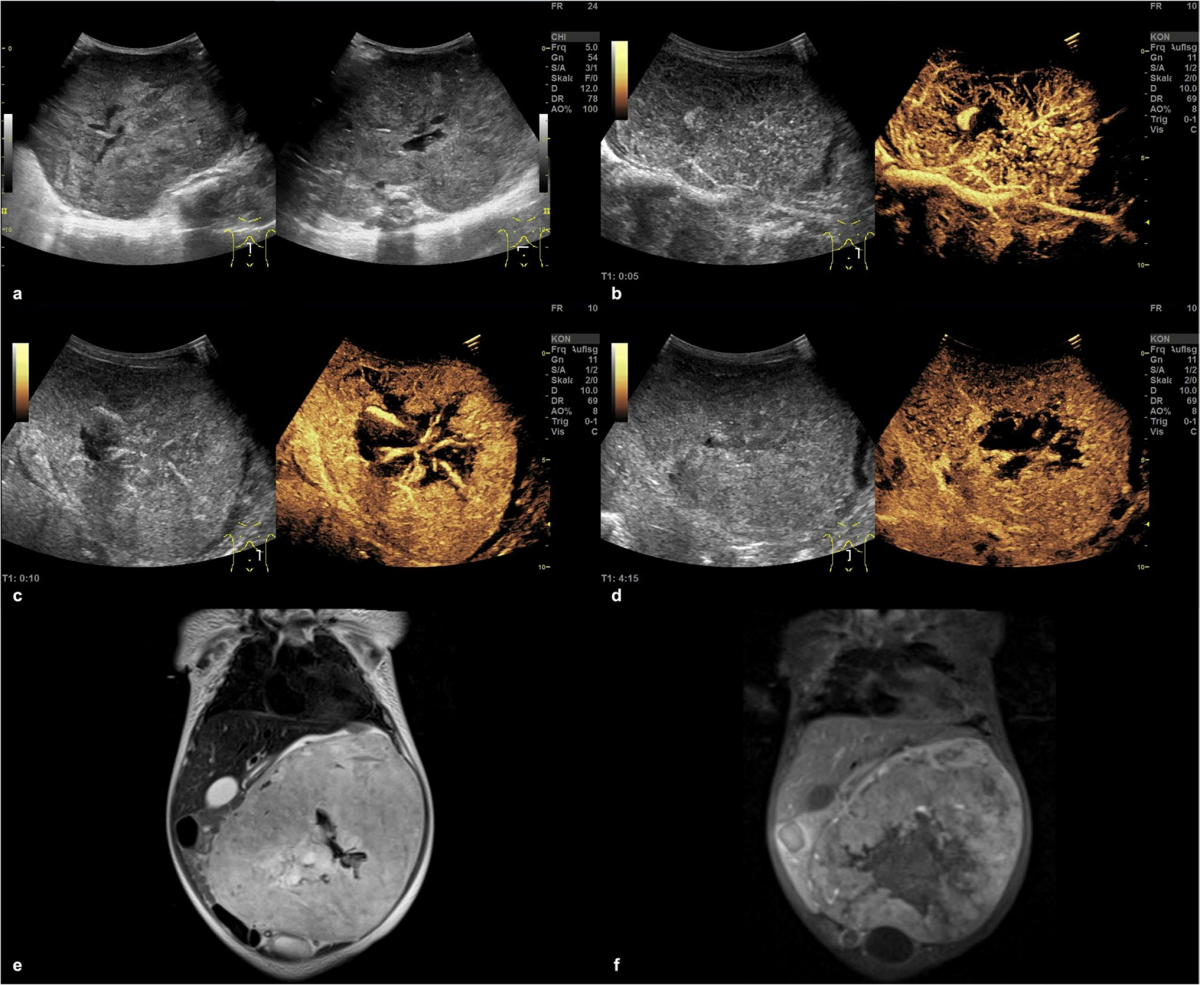
Mesoblastic nephroma in a preterm infant born at 35 ^2^/_7_ weeks’ gestation, examined on the first day of life on the neonatal intensive care unit (weight 3.3 kg, length 50 cm). Huge kidney tumor measuring 11.3 × 13.2 × 7.6 cm (~ 600 mL), arising from the left kidney and crossing the midline. Nephrectomy was performed on the fourth day of life due to abdominal distension and concern for possible malignant progression. **a** Longitudinal and cross-sectional B-Mode scan in the epigastrium of a huge, inhomogeneous kidney tumor reaching out from the left flank across the midline and dorsally until the spine. **b** Longitudinal section through the left flank 5 s post-injection. Early arterial enhancement of the complete length of the abdominal aorta and of the vessel architecture within the mesoblastic nephroma. **c** Early arterial phase after 10 s. Marked rim enhancement of the tumor periphery with hypo-non-enhancement in the center. Delineation of a star-like central vessel configuration. **d** Late phase: still the star-like central vessel configuration is seen in a central area of hypoenhancement. Unlike to normal kidney parenchyma, even after 4 min and 15 s of intermittent scanning, there is still marked contrast enhancement in the tumor. **e** Coronal T2-weighted MRI without fat suppression showing a large left-sided retroperitoneal tumor arising from the left kidney, with central necrosis and hypo-enhanced areas. **f** Coronal post-contrast T1-weighted MRI with fat suppression

Hepatic vascular tumors represented the most frequent findings, accounting for 11 of 23 examinations. These included eight giant congenital hemangiomas (Supplementary File 1 and 2; Fig. 1), one small infantile single high-flow hemangioma, one case of infantile high-flow hepatic hemangiomatosis, and one infantile single low-flow hemangioma (Supplementary File 3 and 4; Fig. 2). In these lesions, CEUS demonstrated early arterial hyperenhancement with progressive centripetal filling (“iris diaphragm phenomenon”) and persistent hyperenhancement without washout. Other tumors included one hepatoblastoma (Fig. 3) and two congenital mesoblastic nephromas (Supplementary File 5; Fig. 4). The renal tumors showed rapid arterial hyperenhancement without washout. In another case, CEUS demonstrated an adrenal mass with early arterial enhancement followed by washout, consistent with adrenal neuroblastoma.

Diffuse hepatic perfusion abnormalities were observed in two neonates with acute liver failure. In these cases, CEUS demonstrated extensive parenchymal hypo- or non-enhancement exceeding in extent the abnormalities visible on B-mode ultrasound. In two additional cases of severe hepatic disease, CEUS demonstrated abnormal hepatic enhancement patterns but did not allow a definitive diagnosis: one examination showed multiple small lesions with early washout throughout the liver, whereas another demonstrated diffuse arterial and portal hyperenhancement without washout. In one infant with multiple cutaneous hemangiomas, CEUS demonstrated an abnormal intrahepatic venous collector, connecting mesenterico-splenic veins to the inferior vena cava, consistent with a congenital portosystemic shunt. CEUS was also used to assess cerebral perfusion in one neonate with neurological deterioration. Enhancement was limited to the basal ganglia and cerebellum, with no detectable enhancement in large parts of the cerebrum. Overall, two CEUS examinations showed normal findings: one hepatic examination and one cerebral perfusion study. Detailed imaging findings and final diagnoses for all cases are provided in Table 2.

**Table 2:** Clinical indication for ultrasound, B-mode/CEUS-findings, and final diagnosis

Case	Clinical indication for ultrasound	B-mode/Doppler findings		CEUS key findings	Final diagnosis
1	Jaundice at 6 days of life with right upper abdominal distension		Large hyperechoic mass in the right hepatic lobe with peripheral hypervascularity and irregular intratumoral vessels	Early arterial hyperenhancement with persistent enhancement and no washout	Hepatoblastoma
2	Prenatally diagnosed rapidly growing liver tumor		Large heterogeneous right hepatic mass extending into the lower abdomen with prominent feeding vessels	Early arterial peripheral hyperenhancement with minimal internal perfusion and persistent peripheral enhancement without washout	Giant congenital hepatic hemangioma (RICH)
3	Postnatal abdominal distension requiring high-flow respiratory support		Large hypervascular mass in the right hepatic lobe with arterial feeders and dilated hepatic veins; mixed echogenicity with few calcifications	Early peripheral arterial enhancement with centripetal fill-in and persistent hyperenhancement without washout; central non-enhancing areas	Giant congenital hepatic hemangioma (RICH)
4	Prenatally detected intraabdominal tumor, likely from the right renal fossa		Large hyperechoic mass arising from the right kidney with moderate Doppler vascularization	Early arterial hyperenhancement with persistent homogeneous enhancement and no washout	Congenital mesoblastic nephroma
5	Marked fetal abdominal enlargement with severe polyhydramnios		Large vascularized mass replacing the left kidney with small calcifications	Early arterial hyperenhancement with central non-enhancing areas and persistent peripheral enhancement without washout	Congenital mesoblastic nephroma
6	Acute liver failure with high-flow respiratory support		Multiple small hyperechoic hepatic nodules without hypervascularity	Initial homogeneous hepatic enhancement followed by early washout in multiple small lesions throughout the liver	Post-necrotic cirrhotic remodelling (liver)
7	Evaluation of acute liver failure		Small liver with irregular nodular surface and a large hyperechoic focal lesion	Arterial and portal hyperenhancement without washout	Neonatal hemochromatosis
8	Incidental finding during routine ultrasound for MMC		Inhomogeneous hyperechoic mass adjacent to the right renal upper pole, separable from the kidney and displacing surrounding organs	Small hyperechoic mass between liver, adrenal gland, and kidney with very early arterial hyperenhancement and early washout	Neuroblastoma (adrenal gland)
9	Evaluation of acute liver failure		Patchy hypoechoic areas (left lobe) and hyperechoic changes (right lobe)	Extensive early and persistent parenchymal hypoenhancement exceeding B-mode findings	Hepatic perfusion disorder
10	Prenatally diagnosed liver tumor		Hypervascular lesion in the right hepatic lobe with large vascular channels draining into dilated hepatic veins and central calcification	Immediate arterial hyperenhancement with prominent tortuous vessels and persistent hyperenhancement without washout; central non-enhancing areas	Giant congenital hepatic hemangioma (RICH)
11	Prenatally diagnosed liver tumor		Hypervascular mixed-echogenic mass in the right hepatic lobe protruding beyond the liver contour	Early arterial peripheral hyperenhancement with centripetal fill-in and no washout; small non-enhancing areas	Infantile single high-flow hemangioma (liver)
12	Prenatal incidental finding initially described as a liver cyst		Solitary slightly hyperechoic hepatic lesion near the right hepatic vein without vascularization	Peripheral nodular arterial enhancement with slow centripetal fill-in and persistent hyperenhancement without washout	Infantile single low-flow hemangioma (liver)
13	Prenatal suspicion of hepatic hemangioma		Focal liver lesion with hypoechoic areas and cystic lacunae, peripheral vascularization	Early arterial rim enhancement with absent central enhancement (no “iris diaphragm phenomenon”)	Giant congenital hepatic hemangioma (RICH)
14	Clinical deterioration with hepatosplenomegaly		Liver at upper normal size with diffuse parenchymal echogenicity changes; no focal lesions	Early hepatic venous enhancement with delayed arterial enhancement and diffuse parenchymal non-enhancement; no focal lesions	Hepatic perfusion disorder
15	Multiple cutaneous hemangiomas		Absent portal vein with a large intrahepatic venous collector receiving inflow from the IVC and mesenterico-splenic veins	Early arterial enhancement with rapid filling of a large intrahepatic venous collector; homogeneous hepatic enhancement without focal lesions; splenic vein inflow with connection to the IVC	Congenital portosystemic shunt (Abernethy malformation)
16	Malformation screening for suspected trisomy 21		Subcapsular hypervascular hepatic mass protruding from the liver surface with tortuous arterial feeders and venous drainage into the middle hepatic vein	Early arterial peripheral-to-central enhancement with persistent hyperenhancement and no washout	Giant congenital hepatic hemangioma (RICH)
17	Multiple cutaneous hemangiomas		Multiple hepatic lesions with hyperechoic rims, hypoechoic centers, and peripheral hypervascularization	Multiple hepatic lesions with peripheral nodular arterial enhancement and centripetal fill-in without washout	Infantile high-flow hepatic hemangiomatosis
18	TGA with seizure		Inhomogeneous basal ganglia with early cystic changes and increased periventricular echogenicity	Normal cerebral perfusion without focal deficits; symmetric enhancement	Normal findings
19	Prenatally diagnosed liver tumor with hemodynamic compromise		Large well-defined right hepatic mass with prominent hepatic artery and dilated hepatic veins	Early peripheral arterial hyperenhancement with large central non-enhancing area and no washout	Giant congenital hepatic hemangioma (RICH)
20	Evaluation of acute liver failure		Homogeneous liver without focal lesions; mild periportal hypoechoic halos	Homogeneous liver enhancement without focal lesions or washout	Normal findings
21	Prenatally diagnosed liver tumor with hemodynamic compromise		Large hyperechoic vascular mass in segment IV with dilated venous lacunae draining into the hepatic veins	Early arterial peripheral enhancement with rapid centripetal fill-in and persistent hyperenhancement without washout	Giant congenital hepatic hemangioma (RICH)
22	Prenatally diagnosed liver tumor		Large hypervascular mass in the left hepatic lobe with tortuous vessels and small calcifications, with dilated hepatic veins	Early peripheral arterial enhancement with centripetal fill-in and persistent hyperenhancement without washout; large non-enhancing areas	Giant congenital hepatic hemangioma (RICH)
23	Neonatal ALL with neurological deterioration		Severely inhomogeneous cerebral parenchyma with loss of gyration and hypoechoic basal ganglia; marked vascular rarefaction with pendular flow	Perfusion in basal ganglia and cerebellum with absent enhancement in large parts of the cerebrum	Cerebral edema, infarction, hemorrhage, possible infiltration (ALL)

*ALL* acute lymphoblastic leukemia, *CEUS* contrast-enhanced ultrasound, *IVC* inferior vena cava, *MMC* myelomeningocele, *RICH* rapid involuting congential hemangioma, *TGA* transposition of the great arteries

### CEUS in comparison with MRI, CT, and biopsy

In 14 of 23 cases (60.9%), CEUS findings were compared with a reference modality (MRI, CT, or biopsy; Supplemental Table 1B and Supplemental Table 2). According to the predefined classification, CEUS was the first modality to establish a definitive diagnosis in ten of these cases (71.4%). In two cases, CEUS provided descriptive findings, with an adrenal neuroblastoma later identified on MRI (case 8) and a giant congenital hepatic hemangioma confirmed by biopsy (case 13). In two additional cases, CEUS made a suspected diagnosis that proved incorrect; biopsy established post-necrotic cirrhotic liver remodelling (case 6) and neonatal hemochromatosis (case 7).

MRI was performed in ten cases: seven yielded presumptive diagnoses (five correct, two incorrect), and three were descriptive. MRI was the first modality to establish a definitive diagnosis in only one case (adrenal neuroblastoma, case 8). CT was performed in three cases, with two descriptive findings and one definitive diagnosis concordant with CEUS (giant congenital hepatic hemangioma, case 3).

Biopsy was performed in eight cases. Three confirmed the CEUS diagnosis directly. In two further cases (giant congenital hepatic hemangioma and congenital mesoblastic nephroma, cases 2 + 5), initial biopsy results differed but were later revised in accordance with CEUS. In the remaining three cases, biopsy established the definitive diagnosis after descriptive or incorrect CEUS findings. In the nine remaining examinations, no independent reference standard was available; CEUS findings were considered consistent with clinical course and treatment response only.

### Safety

No adverse reactions related to UCA administration were observed. Specifically, no anaphylactoid, cardiopulmonary, hemorrhagic, or injection-site complications occurred during or after CEUS examinations.

## Discussion

In this cohort, intravenous CEUS was used as part of routine neonatal imaging, in both term and preterm infants. It was particularly valuable in hepatic vascular tumors, where arteriovenous shunting may lead to significant hemodynamic compromise, including high-output cardiac failure, respiratory deterioration, and consumptive coagulopathy (Kasabach-Merritt phenomenon), and where rapid bedside assessment of potentially unstable neonates is critical for timely decision-making. Beyond these specific indications, CEUS contributed substantially to diagnostic clarification. Among the 14 cases with available reference diagnostics, CEUS findings were consistent with MRI, CT, or biopsy in ten (71.4%); in the remaining nine cases, findings were consistent with clinical course and treatment response only. In several cases, prior imaging was inconclusive, and CEUS provided additional perfusion or lesion characterization, highlighting its role in rapid clinical decision-making. This illustrates that CEUS can complement conventional grayscale and Doppler ultrasound in selected neonatal indications when these techniques remain inconclusive.

An important observation of this study is the feasibility of CEUS under intensive care conditions. More than half of the examinations were performed directly in the NICU, including in ventilated neonates. In neonatal medicine, minimizing stress is a central principle of diagnostic management, and imaging strategies that require transport outside the intensive care environment may expose fragile patients to additional risks [25]. A prospective study of intrahospital neonatal transport reported adverse events in up to one quarter of transports, including cardiorespiratory instability, hypothermia, and equipment-related complications such as accidental extubation [26]. In this context, CEUS offers a practical advantage: dynamic perfusion imaging can be performed at the bedside with continuous monitoring and minimal patient handling. Moreover, CEUS does not involve ionizing radiation in contrast to CT. MRI also avoids radiation but may require sedation or even general anesthesia in some clinical situations. By allowing immediate point-of-care imaging without patient transport, sedation, or ionizing radiation, CEUS may facilitate timely clinical decision-making in critically ill neonates [27].

Published neonatal CEUS experience to date has largely focused on cerebral perfusion imaging. Studies have demonstrated the feasibility of transfontanellar CEUS for assessing neonatal brain perfusion, including quantitative approaches for hypoxic-ischemic injury [13], intraoperative monitoring of cerebral perfusion during neonatal cardiac surgery [22, 23], and correlation with MRI findings in neonatal stroke [15]. In contrast, abdominal CEUS in neonates and particularly in preterm infants has not been described. The present cohort, therefore, extends the reported neonatal CEUS experience to abdominal indications.

Beyond the neonatal setting, previous pediatric studies have described CEUS as a valuable adjunct to CT and MRI [28, 29]. Our findings are consistent with these observations. In this cohort, CEUS frequently provided relevant perfusion and vascular information; however, certain complex conditions did not allow a specific diagnostic classification based on enhancement patterns alone. In such situations, further imaging or histopathological assessment is necessary. Histopathology, therefore, remains the diagnostic reference standard when imaging findings are inconclusive or when therapeutic decisions require tissue confirmation. Previous studies have reported high concordance between CEUS and histopathology, particularly in the evaluation of focal liver and renal lesions in pediatric patients [30, 31]. The somewhat lower agreement observed in our cohort likely reflects the small sample size and the heterogeneous spectrum of pathologies encountered in neonatal and early-life imaging. Two cases with diffuse hepatic disease illustrate specific pitfalls of CEUS interpretation. In one neonate with post-necrotic cirrhotic liver remodelling (case 6), multiple small nodules with early washout were misinterpreted as malignant lesions. Although washout is an established CEUS criterion for malignancy in focal liver lesions, benign conditions including post-necrotic remodelling and regenerative nodules may produce overlapping enhancement patterns—a recognised diagnostic pitfall [32]. In a second case (case 7), B-mode demonstrated a multifocal echogenic hepatic mass with irregular hypervascular architecture, raising strong suspicion for hepatoblastoma. CEUS showed arterial hyperenhancement without washout, but in the context of the compelling B-mode appearance, the overall impression favored malignancy; the final diagnosis established by biopsy was neonatal hemochromatosis. These cases highlight that in diffuse parenchymal disease, integration of B-mode and CEUS findings requires particular caution, and that established enhancement criteria developed for focal lesions have limited applicability in this setting. Larger studies with systematic reference corroboration are needed to establish organ-specific CEUS interpretation criteria in neonates and to further validate the diagnostic patterns described here; the present series may serve as a building block for future comparative work.

### Safety

No immediate or delayed adverse reactions were observed in this cohort following intravenous administration of SonoVue®. Although the absence of complications in a small retrospective series cannot be interpreted as proof of safety, this observation is consistent with large pediatric safety analyses. A systematic review including 4906 intravenous CEUS examinations in 4518 children reported mild reactions in ∼1% and serious adverse events in 0.2% [19]. For comparison, reported acute allergic-like reaction rates for iodinated contrast media used in pediatric CT are approximately 0.18–0.89% [33, 34], while rates for gadolinium-based contrast agents used in MRI are approximately 0.13% of administrations [35]. Nevertheless, as with any contrast agent, appropriate precautions, including emergency equipment and medications to manage anaphylactic reactions, should be available.

### CEUS dosing in neonates and off-label use

In the absence of standardized dosing studies in neonates, UCA administration relies on adapted pediatric or adult recommendations and clinical experience. In this cohort, doses required for adequate enhancement were higher than existing pediatric reference values (e.g., 0.03 ml/kg for Lumason®) [36]. This FDA recommendation is restricted to liver imaging, extrapolated from adult protocols, and not validated in neonates. Common pediatric dosing schemes, such as fixed volumes or simple weight-based formulas, are not directly transferable to neonates as dosage depends on the US system, probe (higher doses needed with linear probes), organ (lower for kidney or spleen than liver), lesion depth, patient-specific variables (age, body size) and circulatory dynamics; even systems and software versions of the same manufacturer may differ. Consequently, empirical weight-based formulas may underestimate the required dose in infants while overestimating it in older children [37]. In clinical practice, dosing therefore requires individual adaptation, and in our study, UCA administration was guided by real-time assessment of enhancement quality and diagnostic interpretability rather than by predefined formulas. Our dosages were comparatively high, as high-frequency linear probes were predominantly used, and there was uncertainty regarding appropriate dosing in neonates prior to the publication of dose recommendations. The dose later recommended by the FDA in 2016 (0.03 ml/kg) results in an absolute dose of 0.06 ml in a 2 kg newborn, which is too low to achieve sufficient enhancement in most organs. Furthermore, due to a learning curve over time, the administered UCA dosage was gradually reduced between 2010 and 2024. All examinations were well tolerated despite higher per-kilogram doses, and no adverse effects occurred.

Given the limited sample size and heterogeneous technical conditions, the doses reported in this study should not be interpreted as dosing recommendations. Prospective and preferably multicenter investigations will be required to establish neonatal-specific dosing strategies and to further characterize the safety profile of intravenous CEUS in this age group. In this context, it should be noted that intravenous CEUS remains off-label for pediatric imaging in Europe, as Sonovue® is approved only for adult use and intravesical application in children. Nevertheless, off-label intravenous use in children is supported by extensive safety data and endorsed by several European radiology societies (e.g., the “European Federation of Societies for Ultrasound in Medicine and Biology,” EFSUMB) as a safe and valuable diagnostic method [38].

### Strengths and limitations

This is the first study to evaluate extracerebral CEUS in a cohort of 23 neonates, including both term and preterm infants. A methodological strength is the inclusion of both stable and critically ill patients, with > 50% of examinations performed in intensive care settings and > 30% classified as urgent assessments. All CEUS examinations followed a standardized protocol and were performed by a single experienced operator, ensuring consistency and internal validity. In addition, detailed case-based documentation of clinical indications, B-mode findings, CEUS enhancement patterns, and final diagnoses is provided in Table 2, allowing transparent assessment of the diagnostic reasoning in individual cases. The inclusion of MRI, CT or biopsy findings in 14 cases enabled comparison of CEUS results with established imaging and pathology standards.

Several limitations must also be acknowledged. The retrospective, single-center design may restrict external validity and limit the generalizability. While the use of a single examiner ensured methodological consistency, it may have introduced observer bias and precluded assessment of inter-observer variability. A further limitation concerns adjudication bias. Since the examiner who performed all CEUS examinations (DF) also participated in the retrospective review, a systematic bias favouring CEUS in the “diagnosis first by” classification cannot be fully excluded. Although AR performed an independent, blinded review without prior knowledge of CEUS findings, with discrepancies resolved by consensus, the possibility of adjudication bias cannot be entirely eliminated. Furthermore, the “diagnosis first by” construct carries an inherent risk of circularity, as the correctness of an interpretation can only be established retrospectively once the final diagnosis is known, potentially favouring the modality whose findings were most consistent with the eventual outcome. Although relatively large for a neonatal CEUS study, the overall sample size remains modest, and indications were heterogeneous, precluding subgroup analysis and limiting conclusions regarding CEUS performance in specific organ systems or pathologies. In particular, 18 of 23 examinations involved hepatic indications, and conclusions regarding CEUS performance in renal, cerebral, and adrenal applications are based on very small case numbers and should be interpreted with caution. Although histopathology represents the diagnostic reference standard, invasive biopsy in neonates is technically demanding due to small anatomical structures and clinical vulnerability. Accordingly, histopathological confirmation was only available in eight cases. In nine cases, no independent reference standard was available, and CEUS findings were evaluated against clinical course and treatment response only, introducing potential verification bias. These cases are reported separately from the 14 cases with available reference diagnostics throughout the manuscript. Prior imaging studies may have influenced the interpretation of CEUS findings. Finally, US systems and transducer technology evolved during the extended study period, which may have affected contrast sensitivity and image quality.

## Conclusion

In summary, this single-center experience suggests that CEUS can be integrated into neonatal imaging practice beyond cerebral applications and may provide clinically relevant diagnostic information predominantly in hepatic conditions, in both term and preterm infants. Its bedside applicability without radiation, sedation, or patient transport may be particularly advantageous in critically ill neonates.

## Supplementary Information

Below is the link to the electronic supplementary material.

Supplementary file1 Giant congenital hepatic hemangioma: Subcostal view of the liver, convex probe, early arterial phase. After the first bubbles appear, prominent peripheral vessels become visible. Gradual centripetal fill-in pattern (“iris diaphragm phenomenon”), with irregularly marginated central areas showing primary non-enhancement, most likely representing necrosis or central hemorrhage. (MP4 45361 KB)

Supplementary file2 Giant congenital hepatic hemangioma: Subcostal oblique view of the liver, linear probe. B-Flow demonstrates markedly widened intralesional vessels within the giant congenital hepatic hemangioma. (MP4 1272 KB)

Supplementary file3 Infantile single low-flow hepatic hemangioma: Subcostal oblique section through the liver, linear probe, early arterial phase. The lesion shows nodular rim enhancement in the early arterial phase. (MP4 5704 KB)

Supplementary file4 Infantile single low-flow hepatic hemangioma: Subcostal oblique section through the liver, linear probe, portal venous phase. Slow centripetal enhancement within the hemangioma from the periphery to the center. Complete fill-in in the late phase without wash out (not shown). (MP4 2870 KB)

Supplementary file5 Mesoblastic nephroma: Longitudinal view in the midline and swept through the huge left sided kidney tumor, convex probe, arterial phase. The outer portions of the mass show hyperenhancement, while the central areas demonstrate primary non-enhancement, consistent with necrosis or intratumoral bleeding. The tumor is highly vascularized, without evidence of washout during an observation period of 3 minutes (not shown). (MP4 20193 KB)

Supplementary Fig. 1 Flowchart of patient inclusion and exclusion for intravenous CEUS in neonates (2010-2024) CEUS: contrast-enhanced ultrasound. (PNG 32.2 KB)

High resolution image (TIF 844 kb)

Supplementary Fig. 2 CEUS-based distribution and classification of diagnosed findings by organ system. *Diagnosis made by CEUS was not correct. In the case of hepatoblastoma, only one of the two diagnoses was incorrect. CEUS: contrast-enhanced ultrasound. (PNG 248 KB)

High Resolution Image (TIFF 4.84 MB)

Supplementary Table 1 (DOCX 36 KB)

Supplementary Table 2 (DOCX 25 KB)

## Data Availability

No datasets were generated or analysed during the current study.

## Abbreviations

CEUS: Contrast-enhanced ultrasound
EFSUMB: European Federation of Societies for Ultrasound in Medicine and Biology
IQR: Interquartile range
NICU: Neonatal intensive care unit
PACS: Picture archiving and communication system
PICC: Peripherally inserted central catheter
SD: Standard deviation
SF_6_: Sulfur hexafluoride
UCA: Ultrasound contrast agent
US: Ultrasound

## Declarations

### Guarantor

The scientific guarantor of this publication is Dr. med. Alexandros Rahn.

### Statistics and biometry

No complex statistical methods were necessary for this paper.

### Study subjects or cohorts overlap

No study subjects or cohorts have been previously reported.

### Methodology

1.) Retrospective, 2.) observational study, 3.) performed at one institution.
