# Supplementary figures and images for: Bubbles in the barely born—contrast-enhanced ultrasound in neonates: a single-center experience
Source: Eur J Pediatr. 2026 Jun 30;185(7):543. doi: 10.1007/s00431-026-07166-0 (PMC13319158; doi:10.1007/s00431-026-07166-0)

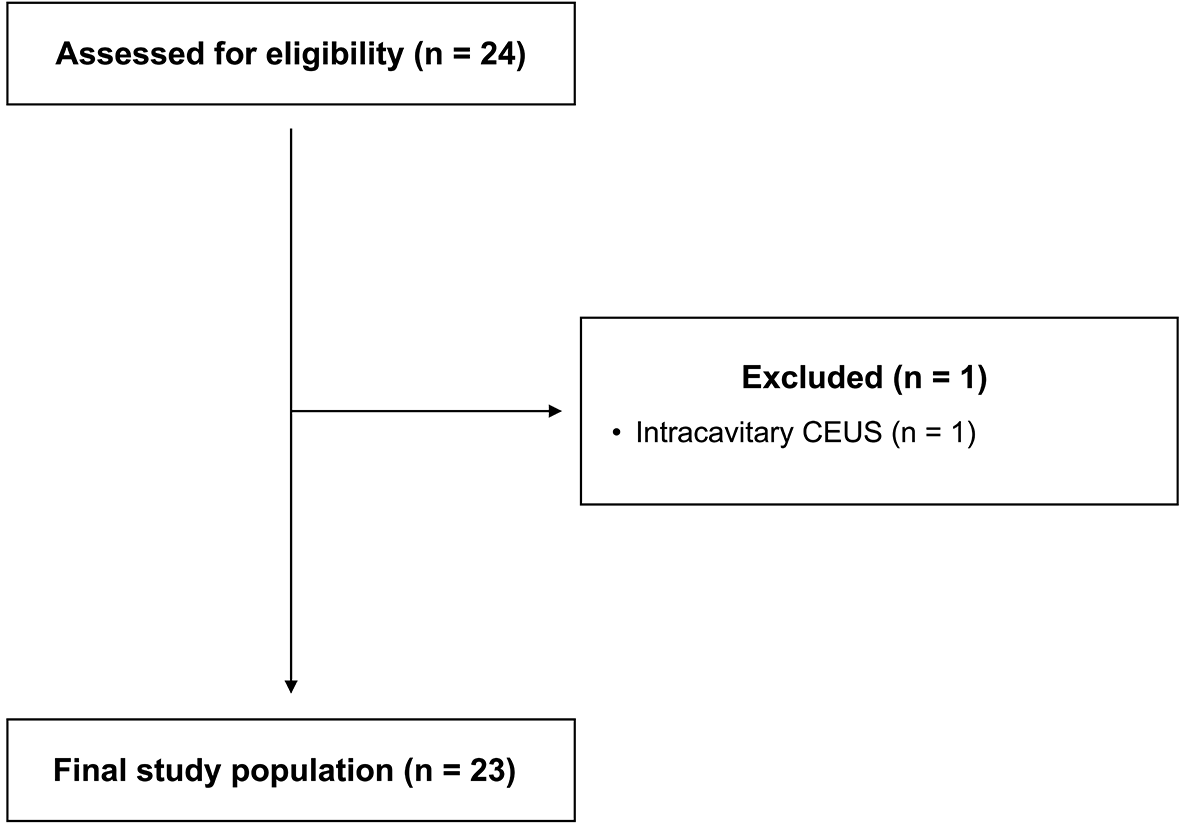

Supplement: Supplementary file 6 — Flowchart of patient inclusion and exclusion for intravenous CEUS in neonates (2010-2024) CEUS: contrast-enhanced ultrasound. (PNG 32.2 KB) [file 431_2026_7166_Fig5_ESM.png]

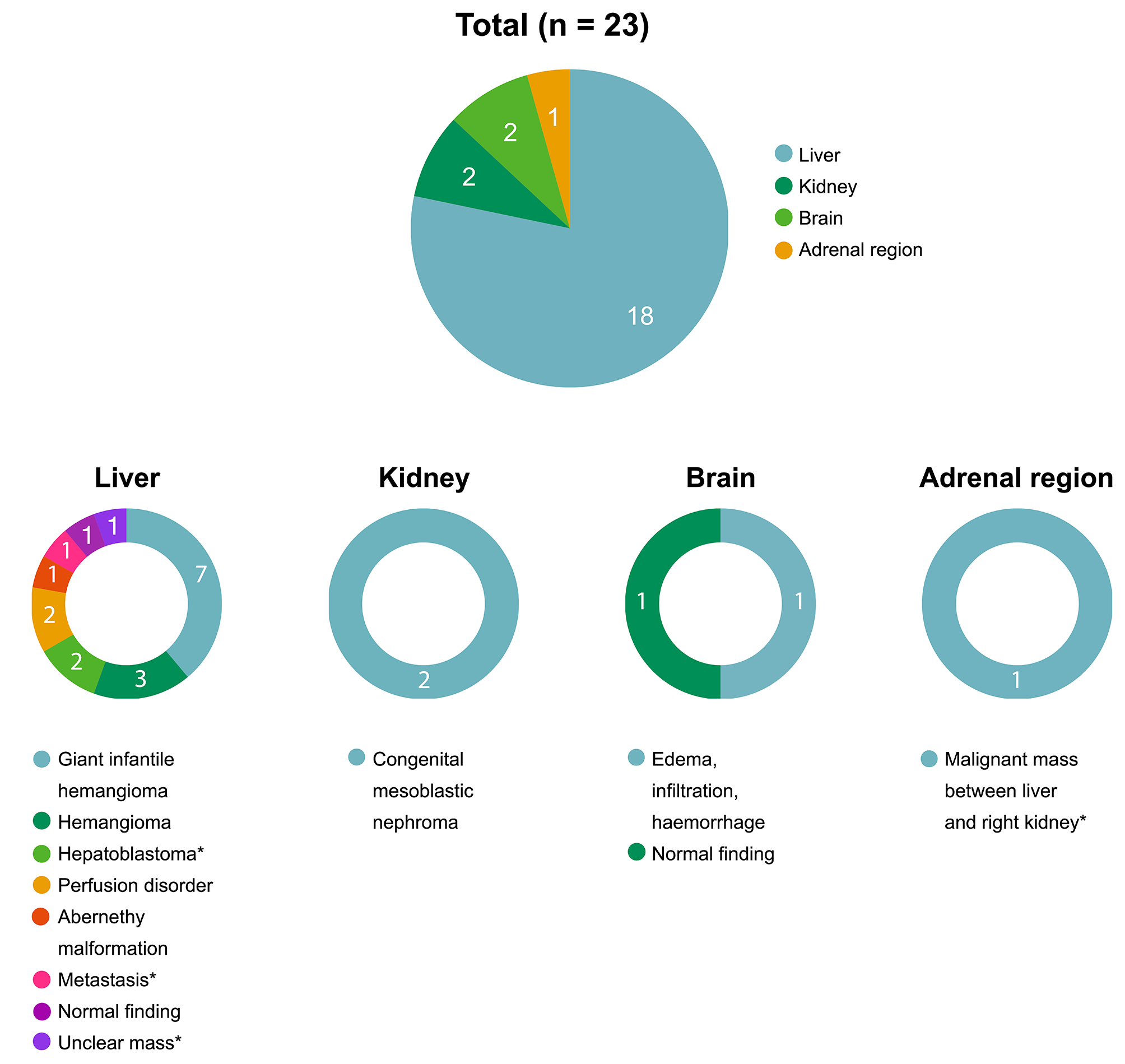

Supplement: Supplementary file 8 — CEUS-based distribution and classification of diagnosed findings by organ system. *Diagnosis made by CEUS was not correct. In the case of hepatoblastoma, only one of the two diagnoses was incorrect. CEUS: contrast-enhanced ultrasound. (PNG 248 KB) [file 431_2026_7166_Fig6_ESM.png]
