# Supplementary material for: Bubbles in the barely born—contrast-enhanced ultrasound in neonates: a single-center experience
Source: Eur J Pediatr. 2026 Jun 30;185(7):543. doi: 10.1007/s00431-026-07166-0 (PMC13319158; doi:10.1007/s00431-026-07166-0)
Supplement: Supplementary file 10 — Supplementary Table 1 (DOCX 36 KB) [file 431_2026_7166_MOESM8_ESM.docx]

**SUPPLEMENTAL TABLE 1A.** Patient characteristics and clinical settings of neonates undergoing contrast-enhanced ultrasound (CEUS).

| Case | Sex | Gestational age (p.m., weeks) | Postnatal age at CEUS (days) | Weight at CEUS (kg) | Organ examined | Setting | Urgent |
| --- | --- | --- | --- | --- | --- | --- | --- |
| 1 | Female | 41 ^1^/_7_ | 9 | 3.46 | Liver | General ward | no |
| 2 | Male | 34 ^1^/_7_ | 23 | 3 | Liver | NICU | no |
| 3 | Male | 39 ^3^/_7_ | 1 | 4.32 | Liver | General ward | no |
| 4 | Male | 35 ^5^/_7_ | 1 | 2.8 | Kidney | NICU | no |
| 5 | Male | 35 ^1^/_7_ | 1 | 3.3 | Kidney | NICU | no |
| 6 | Female | 39 ^6^/_7_ | 26 | 3.6 | Liver | NICU | yes |
| 7 | Male | 39 ^6^/_7_ | 15 | 3.2 | Liver | NICU | no |
| 8 | Female | 39 ^3^/_7_ | 8 | 3.1 | Adrenal region | NICU | no |
| 9 | Female | 38 ^0^/_7_ | 8 | 2.9 | Liver | General ward | no |
| 10 | Male | 38 ^3^/_7_ | 21 | 3.5 | Liver | General ward | no |
| 11 | Male | 41 ^0^/_7_ | 22 | 3.6 | Liver | General ward | yes |
| 12 | Female | 40 ^1^/_7_ | 12 | 2.7 | Liver | General ward | no |
| 13 | Male | 37 ^5^/_7_ | 15 | 2.9 | Liver | General ward | no |
| 14 | Female | 31 ^5^/_7_ | 8 | 1.6 | Liver | NICU | no |
| 15 | Male | 40 ^1^/_7_ | 28 | 4.5 | Liver | General ward | no |
| 16 | Male | 39 ^5^/_7_ | 3 | 3.48 | Liver | General ward | yes |
| 17 | Male | 39 ^6^/_7_ | 27 | 4.6 | Liver | General ward | no |
| 18 | Male | 37 ^4^/_7_ | 25 | 2.73 | Brain | NICU | yes |
| 19 | Male | 34 ^1^/_7_ | 1 | 2.2 | Liver | NICU | yes |
| 20 | Female | 31 ^2^/_7_ | 18 | 1.4 | Liver | NICU | no |
| 21 | Female | 35 ^2^/_7_ | 2 | 2.5 | Liver | NICU | no |
| 22 | Female | 38 ^2^/_7_ | 1 | 3.1 | Liver | NICU | yes |
| 23 | Female | 41 ^3^/_7_ | 23 | 4 | Brain | NICU | yes |

**SUPPLEMENTAL TABLE 1B.** Ultrasound contrast agent (UCA) doses, reference diagnostic and final diagnosis.

| Case | | Number of UCA injections | UCA dose (ml per injection) | UCA dose, weight-adjusted (ml/kg per injection) | Reference diagnostic | Final diagnosis |
| --- | --- | --- | --- | --- | --- | --- |
| 1 | 2 | | 1 / 2 | 0.29 / 0.58 | MRI + CT + Biopsy | Hepatoblastoma |
| 2 | 2 | | 1.2 / 2 | 0.4 / 0.67 | CT + Biopsy | Giant congenital hepatic hemangioma (RICH) |
| 3 | 2 | | 0.8 / 1 | 0.19 / 0.23 | CT | Giant congenital hepatic hemangioma (RICH) |
| 4 | 2 | | 2 / 3 | 0.71 / 1.07 | MRI + Biopsy | Congenital mesoblastic nephroma |
| 5 | 2 | | 1 / 2 | 0.30 / 0.61 | MRI + Biopsy | Congenital mesoblastic nephroma |
| 6 | 2 | | 0.8 / 1 | 0.22 / 0.28 | MRI + Biopsy | Post-necrotic cirrhotic remodelling (liver) |
| 7 | 2 | | 1.4 / 1 | 0.44 / 0.31 | MRI + Biopsy | Neonatal hemochromatosis |
| 8 | 1 | | 2 | 0.65 | MRI | Neuroblastoma (adrenal gland) |
| 9 | 1 | | 1 | 0.34 | MRI | Hepatic perfusion disorder |
| 10 | 2 | | 0.8 / 0.8 | 0.23 / 0.23 | MRI | Giant congenital hepatic hemangioma (RICH) |
| 11 | 1 | | 0.7 | 0.19 | MRI | Infantile single high-flow hemangioma (liver) |
| 12 | 2 | | 1.8 / 0.8 | 0.67 / 0.3 | MRI | Infantile single low-flow hemangioma (liver) |
| 13 | 1 | | 0.5 | 0.17 | Biopsy | Giant congenital hepatic hemangioma (RICH) |
| 14 | 1 | | 1 | 0.63 | Biopsy | Hepatic perfusion disorder |
| 15 | 1 | | 2 | 0.44 | - | Congenital portosystemic shunt (Abernethy malformation) |
| 16 | 2 | | 2 / 1 | 0.57 / 0.29 | - | Giant congenital hepatic hemangioma (RICH) |
| 17 | 2 | | 1 / 0.8 | 0.22 / 0.17 | - | Infantile high-flow hepatic hemangiomatosis |
| 18 | 1 | | 2 | 0.73 | - | Normal findings |
| 19 | 1 | | 1 | 0.45 | - | Giant congenital hepatic hemangioma (RICH) |
| 20 | 2 | | 1.5 / 0.6 | 1.07 / 0.43 | - | Normal findings |
| 21 | 2 | | 0.5 / 1 | 0.2 / 0.4 | - | Giant congenital hepatic hemangioma (RICH) |
| 22 | 1 | | 1.5 | 0.48 | - | Giant congenital hepatic hemangioma (RICH) |
| 23 | 2 | | 2 / 2 | 0.5 / 0.5 | - | Cerebral edema, infarction, hemorrhage, possible infiltration (ALL) |

ALL: acute lymphoblastic leukemia, CT: computed tomography, MRI: magnetic resonance imaging, NICU: neonatal intensive care unit, p.m.: post menstruationem, RICH: rapid involuting congential hemangioma.
