# Supplementary material for: Bubbles in the barely born—contrast-enhanced ultrasound in neonates: a single-center experience
Source: Eur J Pediatr. 2026 Jun 30;185(7):543. doi: 10.1007/s00431-026-07166-0 (PMC13319158; doi:10.1007/s00431-026-07166-0)
Supplement: Supplementary file 11 — Supplementary Table 2 (DOCX 25 KB) [file 431_2026_7166_MOESM9_ESM.docx]

**SUPPLEMENTAL TABLE 2.** Overview of diagnostic methods, final diagnosis, and first identifying modality.

*CT or MRI was performed before CEUS. ^✝︎^Biopsy was performed post mortem. CEUS: contrast-enhanced ultrasound, CT: computed tomography, MRI: magnetic resonance imaging, RICH: rapid involuting congential hemangioma.

| Case | Methods | CEUS | MRI | CT | Biopsy | Final diagnosis | Diagnosis first by |
| --- | --- | --- | --- | --- | --- | --- | --- |
| 1 | CEUS + MRI + CT + Biopsy | Diagnosis | Descriptive | Descriptive | Diagnosis (CEUS confirmed) | Hepatoblastoma | CEUS |
| 2 | CEUS + CT + Biopsy | Diagnosis | - | Descriptive* | Initially incorrect, later CEUS-consistent | Giant congenital hepatic hemangioma (RICH) | CEUS |
| 3 | CEUS + CT | Diagnosis | - | Diagnosis | - | Giant congenital hepatic hemangioma (RICH) | CEUS |
| 4 | CEUS + MRI + Biopsy | Diagnosis | Suspected (correct) | - | Diagnosis (CEUS confirmed) | Congenital mesoblastic nephroma | CEUS |
| 5 | CEUS + MRI + Biopsy | Diagnosis | Descriptive | - | Initially incorrect, later CEUS-consistent | Congenital mesoblastic nephroma | CEUS |
| 6 | CEUS + MRI + Biopsy | Suspected (incorrect) | Descriptive* | - | Diagnosis | Post-necrotic cirrhotic remodelling (liver) | Biopsy |
| 7 | CEUS + MRI + Biopsy | Suspected (incorrect) | Suspected (correct)* | - | Diagnosis | Neonatal hemochromatosis | Biopsy^✝︎^ |
| 8 | CEUS + MRI | Descriptive | Suspected (correct) | - | - | Neuroblastoma (adrenal gland) | MRI |
| 9 | CEUS + MRI | Diagnosis | Suspected (incorrect)* | - | - | Hepatic perfusion disorder | CEUS |
| 10 | CEUS + MRI | Diagnosis | Suspected (correct)* | - | - | Giant congenital hepatic hemangioma (RICH) | CEUS |
| 11 | CEUS + MRI | Diagnosis | Suspected (incorrect)* | - | - | Infantile single high-flow hemangioma (liver) | CEUS |
| 12 | CEUS + MRI | Diagnosis | Suspected (correct)* | - | - | Infantile single low-flow hemangioma (liver) | CEUS |
| 13 | CEUS + Biopsy | Descriptive | - | - | Diagnosis | Giant congenital hepatic hemangioma (RICH) | Biopsy |
| 14 | CEUS + Biopsy | Diagnosis | - | - | Diagnosis (CEUS confirmed) | Hepatic perfusion disorder | CEUS |
